# Association of baseline remnant cholesterol independent of LDL-cholesterol with newly diagnosed diabetes in the Chinese population

**DOI:** 10.17305/bb.2024.11167

**Published:** 2024-10-17

**Authors:** Yulu Yang, Xuehan Li, Jianwu Huang, Jiacheng Wu, Yalei Wang, Hao Chen, Zhihua Qiu, Zihua Zhou

**Affiliations:** 1 Department of Cardiology, Union Hospital, Tongji Medical College, Huazhong University of Science and Technology, Wuhan, China; 2 Hubei Key Laboratory of Biological Targeted Therapy, Union Hospital, Tongji Medical College, Huazhong University of Science and Technology, Wuhan, China; 3 Hubei Engineering Research Center for Immunological Diagnosis and Therapy of Cardiovascular Diseases, Union Hospital, Tongji Medical College, Huazhong University of Science and Technology, Wuhan, China

**Keywords:** Remnant cholesterol, low-density lipoprotein cholesterol, diabetes mellitus, dyslipidemia, prediction model

## Abstract

Remnant cholesterol (RC) is highly regarded in the cardiovascular field; however, its relationship with new-onset diabetes remains unclear. This study aimed to investigate the relationship between RC and the risk of developing diabetes, as well as its interaction with low-density lipoprotein cholesterol (LDL-c). This was a secondary analysis of a retrospective cohort study based on a Chinese population. A multivariate Cox proportional hazards regression was initially employed to assess the relationship between RC and newly diagnosed diabetes. This was followed by a subgroup analysis to explore intergroup heterogeneity. A clinical prediction model was then developed. Finally, the study further analyzed the interactions between LDL-c and RC. After adjusting for confounding factors, RC was significantly associated with an increased risk of diabetes (HR ═ 1.46, 95% confidence interval [CI] 1.36–1.57). Furthermore, this relationship was nonlinear, with an inflection point of 0.48 identified through the piecewise model. Subgroup analysis indicated that the association was more pronounced in individuals under 60 years and those with a body mass index < 24 kg/m^2^ (*P* for interaction ═ 0.0004, <0.0001, respectively). RC proved to be a more effective predictor of diabetes compared to other lipid profiles, and the clinical prediction model was successfully constructed. Notably, individuals in the low LDL-c/high RC group were found to have a 1.41-fold (95% CI 1.281.55) greater risk compared to those in the low LDL-c/low RC group. Significant correlations were observed between baseline RC levels and the risk of new-onset diabetes. Elevated RC was a strong predictor of diabetes risk, irrespective of LDL-c levels.

## Introduction

Diabetes mellitus (DM) is one of the fastest-growing global health emergencies, having reached alarming proportions. The International Diabetes Federation estimates that 537 million adults were living with diabetes in 2021, with this number projected to rise by 46%, reaching approximately 783 million by 2045 [1]. Mortality and disability rates are equally concerning. For type 2 diabetes, the age-standardized death rate has increased to 37.4 per 100,000 adults—a 13.5% rise over the past decade—while disability-adjusted life-years (DALYs) have surged to 1454.5 per 100,000 adults, marking a 26.3% increase [2]. Asian countries bear the highest burden of this disease [3, 4], making diabetes a significant global challenge.

Dyslipidemia plays a critical role in type 2 DM, both as a key component and a major risk factor for macrovascular complications associated with disease progression. Among the lipids of clinical concern, low-density lipoprotein cholesterol (LDL-c) stands out due to its strong association with these complications [5, 6]. In clinical practice, statins have emerged as the first-line treatment for reducing LDL-c levels. Notably, daily treatment with 40 mg of simvastatin has been shown to reduce major vascular events by 22% in diabetic patients compared to a placebo [7]. Furthermore, a meta-analysis of statin therapy in diabetic patients revealed a 9% reduction in all-cause mortality for every mmol/L decrease in LDL-c [8]. Despite the significant reduction in atherosclerotic cardiovascular disease (ASCVD) risk achieved with statins in diabetics, considerable residual risk remains [9–11].

Recent research highlights that triglyceride-rich lipoproteins (TRLs) and their remnants significantly contribute to ASCVD, acting independently of LDL-c [12, 13]. TRLs include chylomicrons from the intestine and very low-density lipoproteins from the liver. Their cholesterol content consists of remnant cholesterol (RC), which includes very low/intermediate-density lipoprotein cholesterol in fasting states and chylomicron remnants in non-fasting states. This expands the range of lipid targets for managing cardiovascular risk [14].

**Figure 1.**
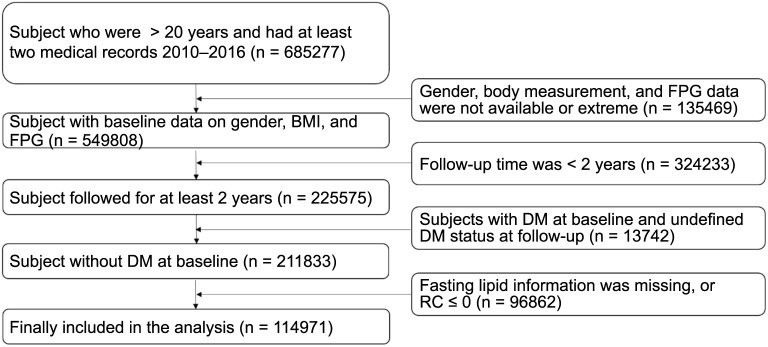
**Flowchart of the screening process for the study population.** RC: Remnant cholesterol; DM: Diabetes mellitus; BMI: Body mass index; FPG: Fasting plasma glucose.

Previous research has established RC as an independent predictor of new-onset diabetes [15, 16] and highlighted its close association with several vascular complications in diabetic patients, including unstable angina pectoris, myocardial infarction, ischemic stroke, coronary revascularization, and cardiovascular death [17, 18]. Additionally, a large-scale, multicenter study in China demonstrated that elevated RC levels were associated with an increased risk of diabetes, even when other conventional lipid levels met guideline-recommended targets [19]. Given the critical clinical importance of LDL-c, there is a compelling need for joint analysis of RC and LDL-c and the development of reliable predictive models to guide medical practice.

In this study, our objective was to explore the relationship between RC and the incidence of new-onset diabetes within the Chinese adult population. We aimed to develop a predictive model to accurately forecast diabetes onset based on RC levels and to investigate the consistency of this association across various levels of LDL-c. By doing so, we hope to provide valuable insights for the primary prevention of diabetes, contributing to more effective management strategies tailored to individual lipid profiles.

## Materials and methods

All data for this study were sourced from the Dryad Digital Repository (https://doi.org/10.5061/dryad.ft8750v), a platform that provides open access to datasets at no cost, ensuring compliance with original authorship rights. This retrospective cohort study utilized data from a comprehensive health screening program conducted across 32 sites in China, retrieved from a computerized database maintained by the Rich Healthcare Group [20]. The study initially included all participants aged 20 and above who attended at least two visits between 2010 and 2016, totaling 685,277 individuals. At baseline, demographic information and fasting blood samples were collected from adults with no prior history of DM. Participants were subsequently monitored for the onset of diabetes, diagnosed either by a fasting plasma glucose (FPG) level of ≥7.00 mmol/L or self-reported diabetes. After excluding participants without recorded lipid levels, 114,971 subjects were included in the final analysis. The research methodology and participant flow are illustrated in Figure 1.

The study collected covariates, including age, gender, systolic and diastolic blood pressures (SBP and DBP), body mass index (BMI), family history of diabetes, smoking status, alcohol consumption, and several biochemical indicators, such as alanine aminotransferase (ALT), aspartate aminotransferase (AST), blood urea nitrogen (BUN), creatinine (CR), FPG, total cholesterol (TC), triglycerides (TG), high-density lipoprotein cholesterol (HDL-c), LDL-c, and RC. Smoking and drinking statuses were classified based on current or former usage. RC was calculated by subtracting LDL-c and HDL-c from TC. Diabetes was diagnosed as described earlier.

**Table 1:** Baseline characteristics of participants

**Characteristic**	**Total**	**Non-DM**	**DM**	***P* value**
N	114,971	112,299	2672	
Age, year	44.14 ± 12.94	43.84 ± 12.80	56.64 ± 12.65	<0.001
Gender, %				<0.001
Male	62,250 (54.14%)	60,369 (53.76%)	1881 (70.40%)	
Female	52721 (45.86%)	51930 (46.24%)	791 (29.60%)	
Family history of DM				<0.001
No	112,374 (97.74%)	109,801 (97.78%)	2573 (96.29%)	
Yes	2597 (2.26%)	2498 (2.22%)	99 (3.71%)	
Smoking status				<0.001
Current	6644 (5.78%)	6387 (5.69%)	257 (9.62%)	
Ever	1322 (1.15%)	1276 (1.14%)	46 (1.72%)	
Never	107,005 (93.07%)	104,636 (93.18%)	2369 (88.66%)	
Drinking status				0.027
Current	862 (0.75%)	831 (0.74%)	31 (1.16%)	
Ever	5483 (4.77%)	5367 (4.78%)	116 (4.34%)	
Never	108,626 (94.48%)	106,101 (94.48%)	2525 (94.50%)	
BMI, kg/m^2^	23.38 ± 3.30	23.31 ± 3.27	26.04 ± 3.43	<0.001
SBP, mmHg	119.50 ± 16.68	119.21 ± 16.51	131.97 ± 18.78	<0.001
DBP, mmHg	74.50 ± 10.97	74.36 ± 10.91	80.58 ± 11.92	<0.001
ALT, U/L	18.20 (13.00–27.60)	18.00 (13.00–27.20)	25.10 (18.00–39.45)	<0.001
AST, U/L	24.09 ± 8.07	24.04 ± 8.02	26.16 ± 9.53	<0.001
BUN, mmol/L	4.69 ± 1.17	4.68 ± 1.16	5.00 ± 1.27	<0.001
CR, umol/L	70.44 ± 15.74	70.38 ± 15.72	73.04 ± 16.36	<0.001
FPG, mmol/L	4.95 ± 0.61	4.92 ± 0.59	5.92 ± 0.71	<0.001
TC, mmol/L	4.80 ± 0.90	4.79 ± 0.89	5.07 ± 0.94	<0.001
TG, mmol/L	1.10 (0.77–1.68)	1.10 (0.76–1.65)	1.71 (1.18–2.50)	<0.001
HDL-c, mmol/L	1.37 ± 0.30	1.37 ± 0.30	1.29 ± 0.29	<0.001
LDL-c, mmol/L	2.77 ± 0.68	2.77 ± 0.68	2.90 ± 0.70	<0.001
RC, mmol/L	0.59 (0.37–0.87)	0.59 (0.37–0.87)	0.81 (0.54–1.13)	<0.001

RC: Remnant cholesterol; LDL-c: Low-density lipoprotein cholesterol; TC: Total cholesterol; HDL-c: High-density lipoprotein cholesterol; SBP: Systolic blood pressure; DBP: Diastolic blood pressure; BMI: Body mass index; ALT: Alanine aminotransferase; AST: Aspartate aminotransferase; BUN: Blood urea nitrogen; CR: Creatinine; FPG: Fasting plasma glucose; TG: Triglycerides; DM: Diabetes mellitus.

All statistical analyses were performed using the R software package, version 4.3.4 (http://www.r-project.org), and EmpowerStats (http://empowerstats.com). Graphs were created using Prism 8.0 (GraphPad Software). The significance threshold was set at *P* < 0.05. Continuous variables were reported as mean ± SD for normally distributed data or as median (interquartile range, IQR) for non-normally distributed data. Categorical variables were expressed as percentages. The analysis began with *t*-tests and chi-square tests to explore differences between diabetic and non-diabetic cohorts, followed by Cox proportional hazard regression to examine the association between RC and the incidence of diabetes, adjusting for potential confounders. Subgroup analyses were conducted to further refine these findings. Generalized additive models, smooth curve fitting, and Kaplan–Meier curves were also used for enhanced data interpretation. The predictive power of RC for new-onset diabetes was evaluated using receiver operating characteristic (ROC) curves, comparing its performance against other lipid metrics. Given the importance of LDL-c, the study population was stratified into four groups based on clinical LDL-c and RC thresholds: low LDL-c and low RC, low LDL-c and high RC, high LDL-c and low RC, and high LDL-c and high RC [19]. The risk of diabetes across these groups was assessed using Kaplan–Meier curves, with the first group serving as the reference.

## Results

### Baseline characteristics of the study population

In the final analysis, 114,971 participants were included (see Table 1). During the follow-up period, 2672 individuals developed diabetes, corresponding to an overall incidence rate of 2.32%. The average age of participants across all study communities was 44.14 ± 12.94 years, with males comprising 54.14% of the population. Individuals who developed diabetes tended to be older, predominantly male, and more likely to have a family history of diabetes. Additionally, they were more likely to smoke and drink compared to those who did not develop diabetes. Furthermore, baseline SBP, DBP, BMI, ALT, AST, BUN, CR, FPG, TC, TG, LDL-c, and RC were higher in the diabetes group, with all differences achieving statistical significance (*P* < 0.05).

### Association of RC with DM

**Figure 2.**
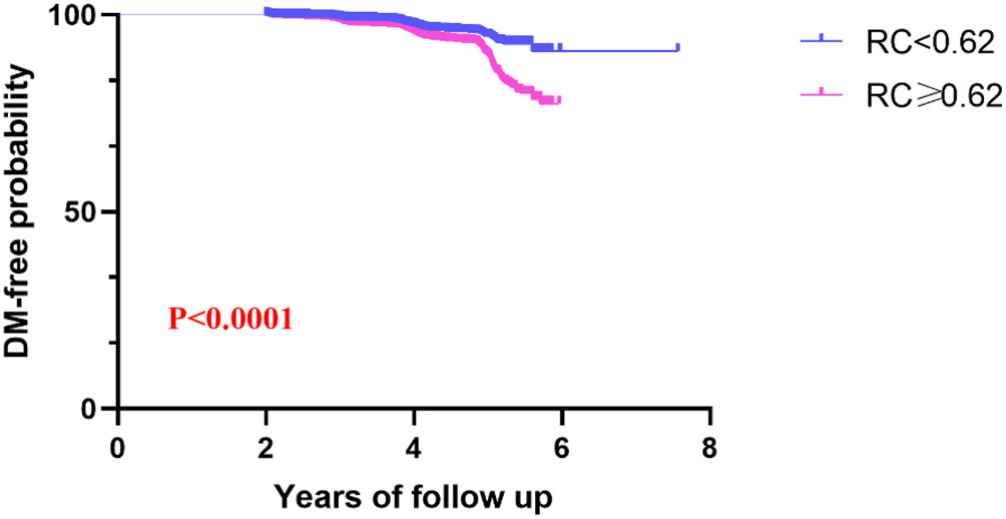
**Kaplan–Meier analysis of diabetes risk according to the RC group.** The *x*-axis represents the follow-up time, and the *y*-axis represents the diabetes-free probability. Each point on the curve indicates the percentage of the study population that remained free of diabetes at that specific time. RC: Remnant cholesterol; DM: Diabetes mellitus.

**Figure 3.**
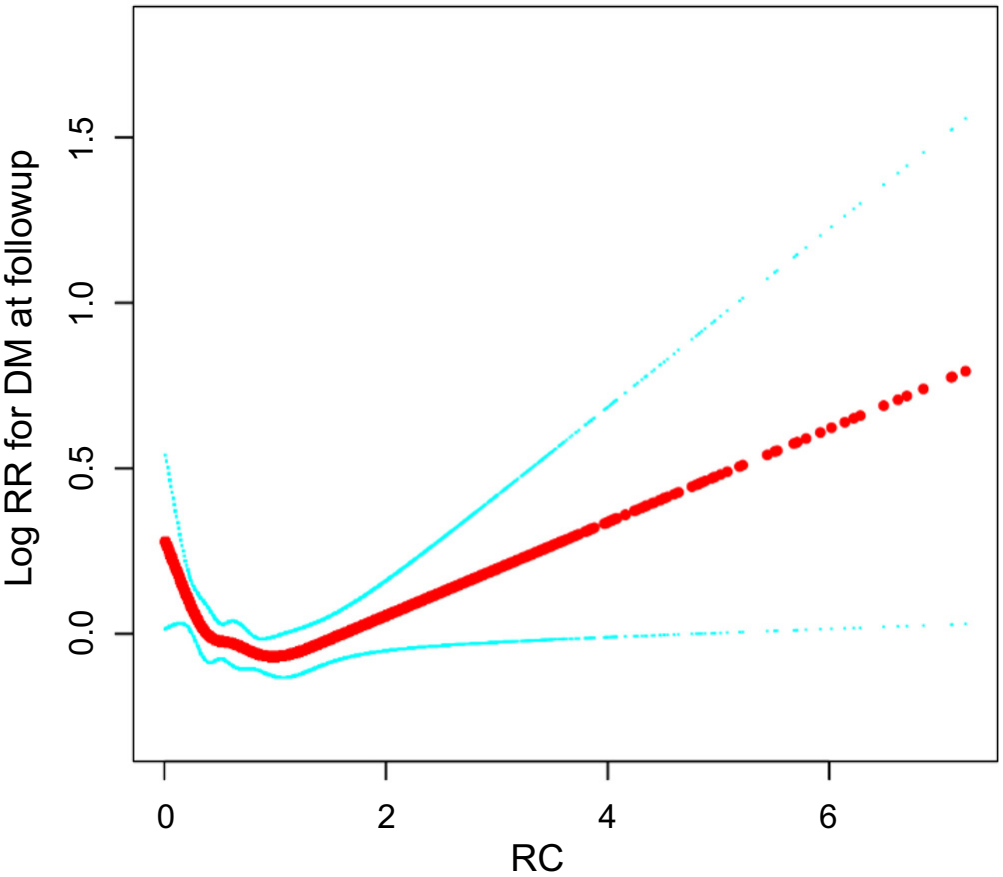
**HR (95% CI) for the non-linear relationship between RC and diabetes.** The *x*-axis represents serum RC levels, while the *y*-axis represents DM risk. The red line indicates the estimate, and the blue shaded area represents the 95% CI. RC: Remnant cholesterol; CI: Confidence interval; DM: Diabetes mellitus.

In the multivariate Cox regression analysis (Table 2), the relationship between RC and the incidence of diabetes was examined both as a continuous and categorical variable, with 0.62 mmol/L used as the cut-off value [21]. Consistent primary findings were observed across the three models, which varied in their levels of adjustment. The association between RC and diabetes was evident in the crude model (HR ═ 2.27, 95% confidence interval [CI]: 2.13, 2.41), Model 1 (HR ═ 1.82, 95% CI: 1.70, 1.95), and the fully adjusted Model 2 (HR ═ 1.46, 95% CI: 1.36, 1.57). In Model 2, each one-unit increase in RC was associated with a 46% increase in diabetes risk. When RC was categorized, individuals with RC ≥ 0.62 mmol/L had a significantly higher risk of developing diabetes (*P* < 0.0001). The temporal pattern of diabetes incidence, displayed in Figure 2, showed a marked increase in cumulative incidence with higher RC levels, using 0.62 mmol/L as the threshold (log-rank *P* < 0.0001). Additionally, Figure 3, incorporating smooth spline fitting, suggested that the relationship between RC and diabetes may not be linear. The inflection point, calculated by the piecewise regression model, was identified at 0.48 mmol/L (Table 3).

**Table 2:** Cox regression analyses for the association between RC and diabetes

**Exposure**	**Crude model** **HR (95% CI, *P* value)**	**Adjust model 1** **HR (95% CI, *P* value)**	**Adjust model 2** **HR (95% CI, *P* value)**
RC	2.27 (2.13, 2.41) < 0.0001	1.82 (1.70, 1.95) < 0.0001	1.46 (1.36, 1.57) < 0.0001
RC < 0.62	Ref	Ref	Ref
RC ≥ 0.62	2.44 (2.24, 2.65) < 0.0001	1.76 (1.62, 1.92) < 0.0001	1.39 (1.27, 1.51) < 0.0001

Crude model: No adjustments made; Adjusted model 1: Adjusted for age and gender; Adjusted model 2: Adjusted for age, gender, BMI, SBP, DBP, smoking, drinking, and family history of diabetes mellitus. RC: Remnant cholesterol; SBP: Systolic blood pressure; DBP: Diastolic blood pressure; BMI: Body mass index; CI: Confidence interval.

### Subgroup analysis

**Figure 4.**
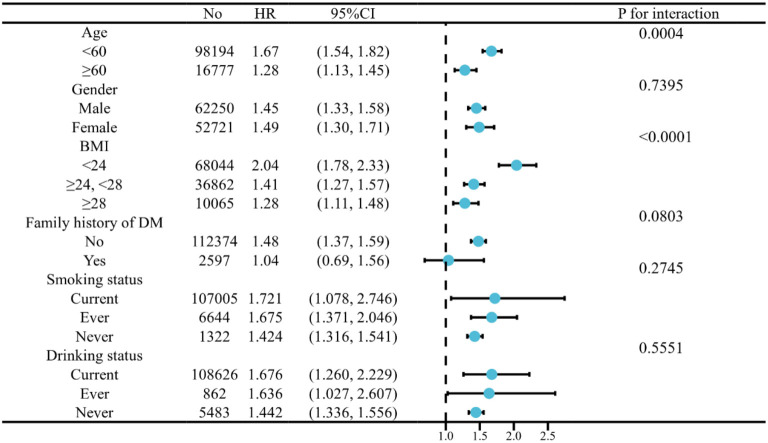
**Stratified analysis of the association between RC and diabetes, based on age, gender, BMI, family history of DM, smoking status, and drinking habits.** RC: Remnant cholesterol; BMI: Body mass index; DM: Diabetes mellitus.

To explore additional risk factors that might influence the relationship between RC and diabetes, as well as to identify potential high-risk populations, we conducted a stratified analysis based on age, gender, BMI, family history of diabetes, smoking status, and drinking status, as detailed in Figure 4. The results revealed that the relationship between RC and the onset of diabetes was notably stronger in individuals under 60 years of age and those with a BMI less than 24. Conversely, the differences in the association between RC and diabetes based on gender, family history, smoking, and drinking status did not reach statistical significance. These findings suggest that age and BMI are more critical factors in the influence of RC on diabetes development.

**Table 3:** Threshold effect analysis of RC and diabetes using piecewise linear regression

**RC**	**Effect size** **(HR)**	**95% CI**	***P* value**
Fitting by the two-piecewise linear model			
Inflection point	0.48		
<0.48	0.47	(0.29, 0.75)	0.0015
>0.48	1.08	(0.99, 1.18)	0.1015
Log-likelihood ratio	0.002		

RC: Remnant cholesterol; CI: Confidence interval.

### Evaluate the accuracy of RC in predicting diabetes

**Figure 5.**
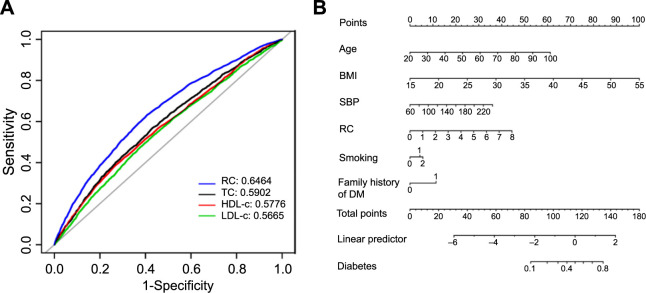
**ROC and nomogram for predicting diabetes.** RC: Remnant cholesterol; LDL-c: Low-density lipoprotein cholesterol; TC: Total cholesterol; HDL-c: High-density lipoprotein cholesterol; ROC: Receiver operating characteristic.

The predictive capabilities of RC, TC, LDL-c, and HDL-c for diabetes risk are detailed in Table 4 and illustrated in Figure 5. The area under the curve (AUC) for RC was significantly superior to those for TC, HDL-c, and LDL-c (*P* < 0.0001). The optimal threshold for RC in predicting the likelihood of developing diabetes was determined to be 0.6750, with a sensitivity of 0.6321 and a specificity of 0.5928. Although the accuracy of RC in predicting future diabetes risk is modest, it outperforms similar lipid indicators and merits attention.

**Table 4:** Predictive potential of TC, HDL-C, LDL-C, and RC in identifying diabetes

	**AUC**	**95% CI low**	**95% CI upp**	**Best threshold**	**Specificity**	**Sensitivity**
TC	0.5902*	0.5792	0.6012	4.8750	0.5721	0.5655
HDL-c	0.5776*	0.5665	0.5888	1.1750	0.7305	0.3877
LDL-c	0.5605*	0.5494	0.5716	2.8350	0.5830	0.5146
RC	0.6464	0.6358	0.6570	0.6750	0.5928	0.6321

RC: Remnant cholesterol; LDL-c: Low-density lipoprotein cholesterol; TC: Total cholesterol; HDL-c: High-density lipoprotein cholesterol; CI: Confidence interval; AUC: Area under the curve.

### Relationship between LDL-c/RC concordance/discordance and diabetes

**Figure 6.**
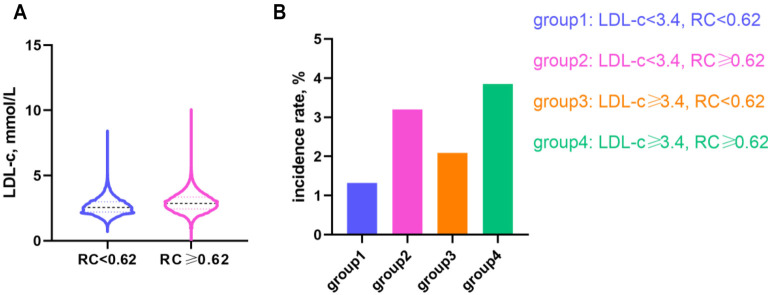
**Distribution of LDL-c and RC, and prevalence rates across different groups.** (A) The violin plot depicts LDL-c levels across different RC groups. The three horizontal lines within the plot represent the lower quartile, median, and upper quartile, respectively. (B) This panel shows the prevalence of diabetes in LDL-c/RC concordance and discordance groups. RC: Remnant cholesterol; LDL-c: Low-density lipoprotein cholesterol.

**Figure 7.**
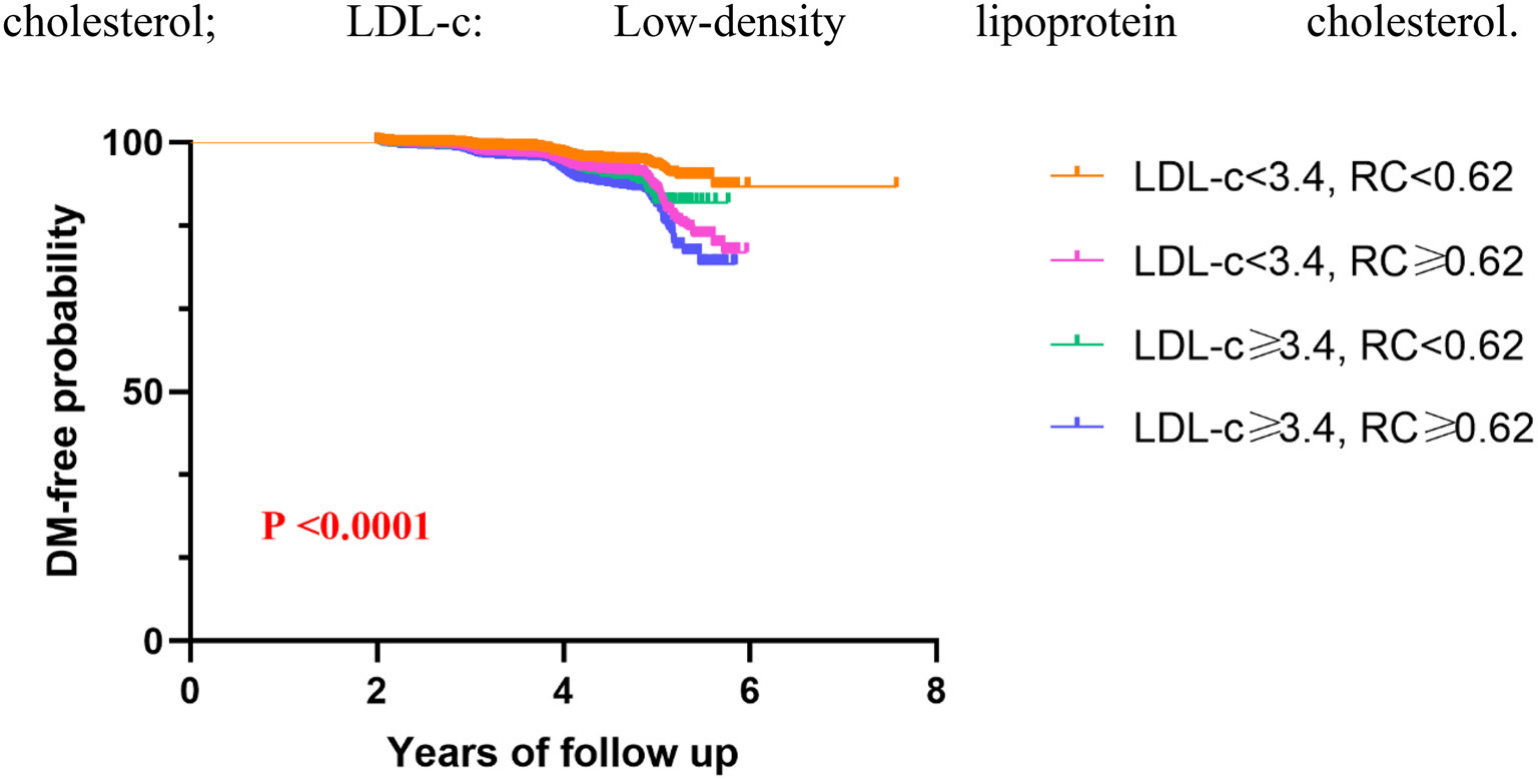
**Kaplan–Meier curves for different groups.** The *x*-axis represents the follow-up time, and the *y*-axis represents the diabetes-free probability. Each point on the curve indicates the percentage of the study population that remained free of diabetes at that specific time. RC: Remnant cholesterol; LDL-c: Low-density lipoprotein cholesterol.

Based on established clinical cut-off points, the study population was categorized into four distinct groups: LDL-c < 3.4 and RC < 0.62, LDL-c < 3.4 and RC ≥ 0.62, LDL-c ≥ 3.4 and RC < 0.62, and LDL-c ≥ 3.4 and RC ≥ 0.62. Figure 6 depicts the distribution of RC and the corresponding prevalence rates of diabetes among these groups. Notably, compared to the group with low LDL-c and low RC, the diabetes risk increased by 1.41-fold (95% CI 1.28–1.55) in the low LDL-c/high RC group and by 1.34-fold (95% CI 1.19–1.52) in the high LDL-c/high RC group, as shown in Table 5. Figure 7 presents Kaplan–Meier curves illustrating diabetes progression over time in these groups. The follow-up period commenced two years after the initial data collection. The prevalence of diabetes was significantly higher in groups with elevated RC, regardless of LDL-c levels. The differences in diabetes onset among these groups were statistically significant, as confirmed by the log-rank test (*P* < 0.0001). This analysis underscores the influential role of RC in diabetes risk, independent of LDL-c levels.

**Table 5:** Relationship between LDL-c/RC concordance/discordance and diabetes

**Exposure**	**Crude model** **HR (95% CI, *P* value)**	**Adjust model 1** **HR (95% CI, *P* value)**	**Adjust model 2** **HR (95% CI, *P* value)**
LDL-c < 3.4, RC < 0.62	Ref	Ref	Ref
LDL-c < 3.4, RC ≥ 0.62	2.48 (2.26, 2.72) < 0.0001	1.81 (1.65, 1.99) < 0.0001	1.41 (1.28, 1.55) < 0.0001
LDL-c ≥ 3.4, RC < 0.62	1.60 (1.33, 1.92) < 0.0001	1.14 (0.94, 1.37) 0.1836	1.01 (0.83, 1.22) 0.9265
LDL-c ≥ 3.4, RC ≥ 0.62	3.00 (2.67, 3.37) < 0.0001	1.77 (1.57, 2.00) < 0.0001	1.34 (1.19, 1.52) < 0.0001

Crude model: No adjustments made; Adjusted model 1: Adjusted for age and gender; Adjusted model 2: Adjusted for age, gender, BMI, SBP, DBP, smoking, drinking, and family history of diabetes mellitus. BMI: Body mass index; RC: Remnant cholesterol; LDL-c: Low-density lipoprotein cholesterol; SBP: Systolic blood pressure; DBP: Diastolic blood pressure; CI: Confidence interval.

## Discussion

This study identified RC as an independent risk factor for the onset of diabetes, outperforming other lipid markers in predictive accuracy. Notably, the impact of RC was especially pronounced in younger individuals (<60 years old) and those with a lower BMI (<24 kg/m^2^). Furthermore, the combined analysis with LDL-c revealed that individuals with low LDL-c and high RC had a greater predisposition to diabetes compared to those with high LDL-c and low RC, suggesting a distinctive role for RC beyond its relationship with LDL-c.

Although research on RC and diabetes is limited, the findings of this study align with existing evidence. In a prospective cohort study conducted in rural China, a 1-SD increase in RC was associated with a 34% higher risk of developing diabetes [22]. Similarly, a single-center study in the general population indicated that for every 1 mmol/L increase in RC levels, the risk of diabetes increased by 1.44-fold [15]. Beyond the development of diabetes itself, RC has also been strongly linked to diabetes-related complications. Data from the Korean National Health Insurance Service showed that in patients newly diagnosed with type 2 diabetes, higher RC levels were associated with a 23.4% increase in kidney complications, despite well-controlled routine lipid values [23]. Additionally, retinopathy, a common complication of diabetes, has also been linked to elevated RC levels. According to a study by Chen et al. [24], higher RC levels were associated with wider retinal arterioles and venules, as well as a higher fractal dimension. Moreover, RC variability has been a consistent predictor of adverse outcomes during follow-up. Research from the National Health and Nutrition Examination Survey (NHANES) demonstrated that in people with diabetes, elevated RC levels were strongly associated with all-cause mortality and cardiovascular death [25]. These findings underscore the complex relationship between RC and diabetes, warranting further in-depth investigation.

Despite the substantial reduction in cardiovascular events attributed to statin therapy, significant residual risk remains. Consequently, many researchers advocate for evaluating LDL-c alongside RC, particularly in the context of cardiovascular disease. For instance, in an analysis involving 9451 patients undergoing revascularization, Zafrir et al. [26] found that RC was a more accurate predictor of myocardial infarction risk, especially when RC levels were inconsistent with LDL-c levels. High myocardial infarction rates were observed when RC levels were at or above the 75th percentile, regardless of LDL-c levels. Similarly, the Beijing Health Management cohort study highlighted RC as a crucial factor in atherosclerosis risk stratification, demonstrating that elevated RC levels increased risk irrespective of LDL-c levels [27]. The increased cardiovascular disease risk associated with RC may stem from its role in atherogenesis and inflammation [28]. RC is more likely than LDL-c to penetrate arterial walls and be taken up by macrophages, accelerating foam cell formation [29]. Mendelian randomization studies have established a causal link between non-fasting RC and low-grade inflammation [30]. Mechanistically, TRL-derived RC can trigger the production of inflammatory cytokines, such as tumor necrosis factor-α and interleukin-1β, promoting leukocyte migration and activation [31]. These findings underscore the pathophysiological importance of RC in both cardiovascular and metabolic diseases.

To the best of our knowledge, there are only a limited number of studies examining the combined effects of RC and LDL-c on diabetes risk. The China Health and Nutrition Survey reported significantly increased risks of diabetes in groups with high RC only and high LDL-c only—304% and 61%, respectively, compared to the low LDL-c/low RC group [32]. However, the limitations inherent to cross-sectional studies must be acknowledged, as they prevent the establishment of a definitive causal relationship. In a large cohort study, Yuan et al. [22] confirmed that individuals with RC levels ≥ 0.56 mmol/L were more than twice as likely to develop type 2 diabetes, independent of their LDL-c levels. Unfortunately, they were unable to develop a prediction model that could be effectively applied in clinical settings. Therefore, our study not only explored the association between RC and diabetes but also constructed a visual prediction model, followed by a joint analysis with LDL-c. This approach is particularly innovative and holds substantial clinical value. Our findings suggest that RC contributes to diabetes development beyond the effects of LDL-c, with the main results visually illustrated using Kaplan–Meier curves.

The precise mechanisms linking RC and dysglycemia are not fully understood, but several potential factors have been proposed. Firstly, atherosclerosis plays a pivotal role in this pathogenic process. RC, known for its strong atherogenic potential, can lead to reduced blood flow and compromised pancreatic function, ultimately resulting in decreased insulin secretion and hyperglycemia [33]. Additionally, atherosclerosis may impair liver function, reducing hepatic glycogen synthesis and contributing to elevated blood glucose levels [34]. Secondly, RC and its oxidation products can directly affect pancreatic beta cells by activating apoptosis signaling pathways, leading to cell death [35]. Thirdly, lipoprotein remnants in RC can interfere with intracellular insulin signaling pathways, potentially binding to insulin receptors or disrupting the phosphorylation and activation of insulin receptor substrates, thereby exacerbating insulin resistance [36, 37].

Furthermore, high RC levels are associated with vascular endothelial dysfunction [38], which increases endothelial cells’ susceptibility to oxidative stress, impairs vasodilation, and affects the delivery of insulin and glucose to tissue cells [39]. Lastly, high RC levels are often linked to systemic inflammatory responses. The production of inflammatory cytokines and reactive oxygen species can contribute to pancreatic beta-cell dysfunction, offering a partial explanation for the relationship between RC and diabetes [40, 41].

One strength of this study was the establishment of a prediction model based on RC and other covariates, enabling physicians to provide primary preventive guidance to high-risk populations. As demonstrated by Sheng et al. [42], the predictive value of RC for future diabetes was significantly higher than that of conventional lipid parameters. Other researchers have used various indicators to forecast diabetes risk, such as unconventional body measurements and laboratory characteristics, but their predictive power did not exceed the comprehensive prediction model in the current study [43–45]. This further underscores the significant role of RC. Interestingly, the impact of RC on diabetes risk was particularly pronounced in adults under 60 and those with a BMI < 24. This finding aligns with previous research showing a stronger association between RC and myocardial infarction risk in younger individuals with type 2 diabetes [46]. On the one hand, the metabolic systems of younger people tend to have greater plasticity, making their metabolic regulation mechanisms more easily disturbed by elevated RC levels. Additionally, poor lifestyle habits, such as unhealthy diets, lack of exercise, and insufficient sleep, are more common among younger adults, who may also pay less attention to their health. These factors, in combination with high RC levels, could increase the risk of developing diabetes. On the other hand, individuals with low BMI may have different metabolic patterns. Some may exhibit the phenomenon of being “metabolically unhealthy lean,” meaning that despite having normal weight, they experience metabolic problems, such as insulin resistance and dyslipidemia. In such cases, elevated RC may be more likely to trigger diabetes development. Moreover, people with low BMI may lack certain nutrients due to dietary restrictions, which can affect metabolic regulation and make them more sensitive to changes in RC levels.

As recognized in the latest guidelines for the management of dyslipidemia [21], current clinical lipid-lowering therapies are still primarily focused on LDL-c. However, our findings revealed that regardless of LDL-c levels, individuals with elevated RC had a higher likelihood of developing diabetes. Therefore, we suggest that, in addition to LDL-c, RC may be another lipid marker requiring greater clinical attention. It seems necessary to report and monitor RC levels during clinical examinations. For individuals at high risk of diabetes, regular RC testing could be considered for early diabetes risk assessment. For patients who already have diabetes, RC can be measured regularly to assess the risk of cardiovascular complications, depending on the patient’s condition and the physician’s recommendations. Furthermore, combining RC with traditional risk factors to build a diabetes risk assessment model could help classify patients into different risk categories. For high-risk individuals, more aggressive interventions could be implemented, such as strengthening lifestyle modifications, regular blood glucose monitoring, and medication if necessary. Although current guidelines do not specifically recommend strategies for lowering RC, several therapeutic trials are underway exploring treatments for elevated RC. These studies are primarily investigating the efficacy of statins [47], fibrates [48], and omega-3 fatty acids [49]. However, no consensus has been reached regarding the best approach.

In addition, advancements in genetic research hold promise for the development of targeted drug therapies. This emerging field could lead to more precise and effective treatments for managing elevated RC, presenting an exciting prospect for the future. It is important to acknowledge several limitations of this study. Firstly, the sample consisted exclusively of participants from China. While this provided robust representation of the Chinese population, extrapolating these findings to other ethnic or demographic groups should be done with caution. Additionally, our study did not differentiate between type 1 and type 2 diabetes. Since type 2 diabetes accounts for approximately 95% of all diabetes cases, our findings are likely more representative of type 2 diabetes. Thirdly, while we adjusted for several key covariates, the possibility of confounding factors, such as dietary habits, medication use, occupational exposures, and environmental influences, remains. Finally, our analysis was based on baseline RC levels, which may vary due to changes in medication or lifestyle. Therefore, the dynamic fluctuations of RC warrant further investigation to better understand its long-term implications.

## Conclusion

Our study demonstrated a clear upward trend in diabetes risk associated with increasing RC levels, particularly among younger individuals and those with a lower BMI. These findings underscore the potential for developing a new diabetes risk prediction model that incorporates RC as a key factor. This approach could enhance our understanding and management of diabetes risk, paving the way for more personalized and effective preventive strategies.

## References

[1] Sun H, Saeedi P, Karuranga S, Pinkepank M, Ogurtsova K, Duncan BB (2022). IDF diabetes atlas: Global, regional and country-level diabetes prevalence estimates for 2021 and projections for 2045. Diabetes Res Clin Pract.

[2] Cousin E, Schmidt MI, Ong KL, Lozano R, Afshin A, Abushouk AI (2022). Burden of diabetes and hyperglycaemia in adults in the Americas, 1990-2019: a systematic analysis for the global burden of disease study 2019. Lancet Diabetes Endocrinol.

[3] Yang JJ, Yu D, Wen W, Saito E, Rahman S, Shu XO (2019). Association of diabetes with all-cause and cause-specific mortality in Asia: a pooled analysis of more than 1 million participants. JAMA Netw Open.

[4] Mettananda KC, Mettananda S (2024). Burden of disease scenarios for 204 countries and territories, 2022-2050: a forecasting analysis for the global burden of disease study 2021. Lancet (London, England).

[5] Haffner SM (1998). Management of dyslipidemia in adults with diabetes. Diabetes Care.

[6] Betteridge DJ (2011). Lipid control in patients with diabetes mellitus. Nat Rev Cardiol.

[7] Collins R, Armitage J, Parish S, Sleigh P, Peto R (2003). MRC/BHF Heart Protection Study of cholesterol-lowering with simvastatin in 5963 people with diabetes: a randomised placebo-controlled trial. Lancet (London, England).

[8] Kearney PM, Blackwell L, Collins R, Keech A, Simes J, Peto R (2008). Efficacy of cholesterol-lowering therapy in 18,686 people with diabetes in 14 randomised trials of statins: a meta-analysis. Lancet (London, England).

[9] Stahel P, Xiao C, Hegele RA, Lewis GF (2018). The atherogenic dyslipidemia complex and novel approaches to cardiovascular disease prevention in diabetes. Can J Cardiol.

[10] Goldberg RB (2022). Dyslipidemia in diabetes: when and how to treat? Endocrinol Metab. Clin North Am.

[11] Gomez-Delgado F, Raya-Cruz M, Katsiki N, Delgado-Lista J, Perez-Martinez P (2024). Residual cardiovascular risk: When should we treat it?. Eur J Intern Med.

[12] Chapman MJ, Ginsberg HN, Amarenco P, Andreotti F, Borén J, Catapano AL (2011). Triglyceride-rich lipoproteins and high-density lipoprotein cholesterol in patients at high risk of cardiovascular disease: evidence and guidance for management. Eur Heart J.

[13] Quispe R, Martin SS, Michos ED, Lamba I, Blumenthal RS, Saeed A (2021). Remnant cholesterol predicts cardiovascular disease beyond LDL and ApoB: a primary prevention study. Eur Heart J.

[14] Varbo A, Nordestgaard BG (2017). Remnant lipoproteins. Curr Opin Lipidol.

[15] Xie G, Zhong Y, Yang S, Zou Y (2021). Remnant cholesterol is an independent predictor of new-onset diabetes: a single-center cohort study. Diabetes, Metab Syndr Obes.

[16] Li B, Liu Y, Zhou X, Gu W, Mu Y (2024). Remnant cholesterol, but not other traditional lipids or lipid ratios, is independently and positively related to future diabetes risk in Chinese general population: a 3 year cohort study. J Diabetes Investig.

[17] Yu D, Wang Z, Zhang X, Qu B, Cai Y, Ma S (2021). Remnant cholesterol and cardiovascular mortality in patients with type 2 diabetes and incident diabetic nephropathy. J Clin Endocrinol Metab.

[18] Cao YX, Zhang HW, Jin JL, Liu HH, Zhang Y, Gao Y (2020). The longitudinal association of remnant cholesterol with cardiovascular outcomes in patients with diabetes and pre-diabetes. Cardiovasc Diabetol.

[19] Li B, Zhou X, Wang W, Gao Z, Yan L, Qin G (2023). Remnant cholesterol is independently associated with diabetes, even if the traditional lipid is at the appropriate level: a report from the REACTION study. J Diabetes.

[20] Chen Y, Zhang XP, Yuan J, Cai B, Wang XL, Wu XL (2018). Association of body mass index and age with incident diabetes in Chinese adults: a population-based cohort study. BMJ Open.

[21] Mach F, Baigent C, Catapano AL, Koskinas KC, Casula M, Badimon L (2020). 2019 ESC/EAS Guidelines for the management of dyslipidaemias: lipid modification to reduce cardiovascular risk. Eur Heart J.

[22] Yuan L, Liu J, Huang Z, Zhao Y, Feng Y, Yang X (2023). Elevated remnant cholesterol increase 6-year type 2 diabetes mellitus onset risk. Clin Chim Acta.

[23] Jang SY, Kang M, Song E, Jang A, Choi KM, Baik SH (2024). Remnant cholesterol is an independent risk factor for the incidence of chronic kidney disease in newly-diagnosed type 2 diabetes: a nationwide population-based study. Diabetes Res Clin Pract.

[24] Chen S, Xu Y, Chen B, Lin S, Lu L, Cheng M (2024). Remnant cholesterol is correlated with retinal vascular morphology and diabetic retinopathy in type 2 diabetes mellitus: a cross-sectional study. Lipids Health Dis.

[25] Pan D, Xu L, Zhang LX, Shi DZ, Guo M (2024). Associations between remnant cholesterol levels and mortality in patients with diabetes. World J Diabetes.

[26] Zafrir B, Khoury R, Saliba W (2023). Remnant cholesterol and risk of myocardial infarction in patients with coronary artery disease undergoing revascularization. J Clin Lipidol.

[27] Wu Z, Wang J, Zhang H, Pan H, Li Z, Liu Y (2023). Longitudinal association of remnant cholesterol with joint arteriosclerosis and atherosclerosis progression beyond LDL cholesterol. BMC Med.

[28] Sandesara PB, Virani SS, Fazio S, Shapiro MD (2019). The forgotten lipids: triglycerides, remnant cholesterol, and atherosclerotic cardiovascular disease risk. Endocr Rev.

[29] Wang K, Wang R, Yang J, Liu X, Shen H, Sun Y (2022). Remnant cholesterol and atherosclerotic cardiovascular disease: metabolism, mechanism, evidence, and treatment. Front Cardiovasc Med.

[30] Varbo A, Benn M, Tybjærg-Hansen A, Nordestgaard BG (2013). Elevated remnant cholesterol causes both low-grade inflammation and ischemic heart disease, whereas elevated low-density lipoprotein cholesterol causes ischemic heart disease without inflammation. Circulation.

[31] Shin HK, Kim YK, Kim KY, Lee JH, Hong KW (2004). Remnant lipoprotein particles induce apoptosis in endothelial cells by NAD(P)H oxidase-mediated production of superoxide and cytokines via lectin-like oxidized low-density lipoprotein receptor-1 activation: prevention by cilostazol. Circulation.

[32] Hu X, Liu Q, Guo X, Wang W, Yu B, Liang B (2022). The role of remnant cholesterol beyond low-density lipoprotein cholesterol in diabetes mellitus. Cardiovasc Diabetol.

[33] Levy BI, Schiffrin EL, Mourad JJ, Agostini D, Vicaut E, Safar ME (2008). Impaired tissue perfusion: a pathology common to hypertension, obesity, and diabetes mellitus. Circulation.

[34] Chirinos JA, Segers P, Hughes T, Townsend R (2019). Large-artery stiffness in health and disease: JACC state-of-the-art review. J Am Coll Cardiol.

[35] Lu X, Liu J, Hou F, Liu Z, Cao X, Seo H (2011). Cholesterol induces pancreatic β cell apoptosis through oxidative stress pathway. Cell Stress Chaperones.

[36] Saltiel AR, Kahn CR (2001). Insulin signalling and the regulation of glucose and lipid metabolism. Nature.

[37] Sabapathy T, Helmerhorst E, Ellison G, Bridgeman SC, Mamotte CD (2022). High-fat diet induced alterations in plasma membrane cholesterol content impairs insulin receptor binding and signalling in mouse liver but is ameliorated by atorvastatin. Biochim Biophys Acta Mol Basis Dis.

[38] Yang PT, Li Y, Wang JG, Zhang LJ, Yang SQ, Tang L (2023). The association of remnant cholesterol with endothelial dysfunction and subclinical atherosclerosis in a check-up population in China. J Atheroscler Thromb.

[39] Schnitzler JG, Dallinga-Thie GM, Kroon J (2019). The role of (modified) lipoproteins in vascular function: a duet between monocytes and the endothelium. Curr Med Chem.

[40] Böni-Schnetzler M, Meier DT (2019). Islet inflammation in type 2 diabetes. Semin Immunopathol.

[41] Di Marco E, Jha JC, Sharma A, Wilkinson-Berka JL, Jandeleit-Dahm KA, de Haan JB (2015). Are reactive oxygen species still the basis for diabetic complications?. Clin Sci (Lond).

[42] Sheng G, Kuang M, Yang R, Zhong Y, Zhang S, Zou Y (2022). Evaluation of the value of conventional and unconventional lipid parameters for predicting the risk of diabetes in a non-diabetic population. J Transl Med.

[43] Yang J, Wang F, Wang J, Han X, Hu H, Yu C (2018). Using different anthropometric indices to assess prediction ability of type 2 diabetes in elderly population: a 5 year prospective study. BMC Geriatr.

[44] Xu T, Yu D, Zhou W, Yu L (2022). A nomogram model for the risk prediction of type 2 diabetes in healthy eastern China residents: a 14-year retrospective cohort study from 15,166 participants. EPMA J.

[45] López-Gómez SA, González-López BS, Scougall-Vilchis RJ, Márquez-Corona ML, Minaya-Sánchez M, Navarrete-Hernández JJ (2022). Factors associated with self-report of type 2 diabetes mellitus in adults seeking dental care in a developing country. Int J Environ Res Public Health.

[46] Huh JH, Han KD, Cho YK, Roh E, Kang JG, Lee SJ (2022). Remnant cholesterol and the risk of cardiovascular disease in type 2 diabetes: a nationwide longitudinal cohort study. Cardiovasc. Diabetol.

[47] Miller PE, Martin SS, Joshi PH, Jones SR, Massaro JM, D’Agostino RB (2016). Pitavastatin 4 mg provides significantly greater reduction in remnant lipoprotein cholesterol compared with pravastatin 40 mg: results from the short-term phase IV PREVAIL US trial in patients with primary hyperlipidemia or mixed dyslipidemia. Clin Ther.

[48] Tsunoda F, Asztalos IB, Horvath KV, Steiner G, Schaefer EJ, Asztalos BF (2016). Fenofibrate, HDL, and cardiovascular disease in type-2 diabetes: the DAIS trial. Atherosclerosis.

[49] Sezai A, Unosawa S, Taoka M, Osaka S, Obata K, Kanno S (2019). Long-term comparison of ethyl icosapentate vs. omega-3-acid ethyl in patients with cardiovascular disease and hypertriglyceridemia (DEFAT trial). Circ J.

